# Combined Transcriptomic and Lipidomic Analysis Reveals Dysregulated Genes Expression and Lipid Metabolism Profiles in the Early Stage of Fatty Liver Disease in Rats

**DOI:** 10.3389/fnut.2021.733197

**Published:** 2021-09-17

**Authors:** Ruina Zhai, Lei Feng, Yu Zhang, Wei Liu, Shengli Li, Zhiyong Hu

**Affiliations:** ^1^College of Animal Science, Xinjiang Agricultural University, Urumqi, China; ^2^Ruminant Nutrition and Physiology Laboratory, College of Animal Science and Technology, Shandong Agricultural University, Taian, China; ^3^State Key Laboratory of Animal Nutrition, College of Animal Science and Technology, China Agricultural University, Beijing, China

**Keywords:** non-alcoholic fatty liver disease, rats, lipidomic, transcriptomic, fatty acids

## Abstract

Non-alcoholic fatty liver disease develops from simple steatosis to non-alcoholic steatohepatitis (NASH), which then potentially develops into liver cirrhosis. It is a serious threat to human health. Therefore, investigating the formation and development mechanism of non-alcoholic fatty liver disease (NAFLD) is of great significance. Herein, an early model of NAFLD was successfully established by feeding rats with a high-fat and choline-deficient diet. Liver tissue samples were obtained from rats in the fatty liver model group (NAFL) and normal diet control group (CON). Afterward, transcriptome and lipidomic analysis was performed. Transcriptome results revealed that 178 differentially expressed genes were detected in NAFL and CON groups. Out of which, 105 genes were up-regulated, 73 genes were downregulated, and 8 pathways were significantly enriched. A total of 982 metabolites were detected in lipidomic analysis. Out of which 474 metabolites were significantly different, 273 were up-regulated, 201 were downregulated, and 7 pathways were significantly enriched. Based on the joint analysis, 3 common enrichment pathways were found, including cholesterol metabolism and fat digestion and absorption metabolic pathways. Overall, in the early stage of NAFLD, a small number of genetic changes caused a strong response to lipid components. The strongest reflection was glycerides and glycerophospholipids. A significant increase in fatty acid uptake accompanied by cholesterol metabolism is the most prominent metabolic feature of the liver in the early stage of NAFLD. In the early stage of fatty liver, the liver had shown the characteristics of NASH.

## Introduction

Non-alcoholic fatty liver disease is one of the most common chronic liver diseases (1), one fourth of the global population is estimated to have it (2). Non-alcoholic fatty liver disease (NAFLD) is generally divided into simple steatosis (NAFL) and non-alcoholic steatohepatitis (NASH) (generally developed from NAFL) (3, 4), which may lead to liver cirrhosis, hepatic failure and hepatocellular carcinoma (5, 6). NAFLD is already the fastest growing cause of hepatocellular carcinoma in many countries (2). Urgent measures that understand the occurrence of NAFLD are necessary. The study focused on the early stage of NAFLD because it is important to know what happens in the liver when NAFLD occurs. Through a combined transcriptomic and lipidomic analysis of liver tissue, the occurrence of NAFLD can be better understood.

Lipid accumulation in hepatocytes is the initial and pre-requisite step in the development of NAFLD (7–9). The causes of lipid deposition in hepatocytes are divided into the following points: (a) increased sources of fatty acids (FAs), excessive entry of FAs from diet or adipose tissue into the liver, and/or increased *de novo* lipogenesis (DNL), (b) reduced lipid consumption, reduced fat oxidation and/or reduced output of triglycerides (TGs) in the form of very-low-density lipoproteins (4, 10). Based on the two-hit hypotheses, the first hit originates from an accumulation of more than 5% hepatic steatosis, during which insulin resistance becomes a pathogenic factor, which increases the vulnerability of the liver to a second hit and develops into NASH (11). In the theory of multiple-hit hypotheses, insulin resistance is also considered a vital factor causing further development of NAFLD (12). Recent studies indicate that FAs patterns and phospholipid composition of liver samples from patients with NAFLD have significantly been altered (13). It is also believed that lipid metabolism disorder regulates the occurrence and development of NAFLD. Additionally, regardless of the hit source, the response of the liver to extrahepatic stimulation is modulated by transcriptional regulation (14). Therefore, the joint lipidomic and transcriptomic analysis of NAFL accurately reflects its actual situation.

High-fat diet, western diet, methionine- and choline-deficient diet, choline-deficient and L-amino acid-deficient diet, etc. are the common diet-induced models (14). Choline deficiency and high-fat diets have a satisfactory impact on the induction of the NAFLD model. Thus, the study combined choline deficiency and a high-fat diet to trigger early fatty liver models in rats (14). In addition, to explain NAFL occurrence, lipidomic and transcriptomic analyses of the rat liver were conducted.

## Materials and Methods

### Animals and Diets

The Animal Welfare and Health Committee of Shandong Agricultural University approved the animal housing and handling procedures. The experiment used 14 8-week-old male Sprague–Dawley rats fed with a standard diet with 1 week of acclimatization. The rats were later randomly assigned into the study (NAFL group, *n* = 8) and control groups (CON group, *n* = 6). The CON group were fed with a normal diet (i.e., 4.3% fat, 10% kcal from fat, 70% kcal from carbohydrate, 20% kcal from protein, D12450B), whereas the NAFL group was supplied with a high-fat diet, without choline (i.e., 23.7% fat, 44.9% kcal from fat, 35.1% kcal from carbohydrate, 20% kcal from protein, D05010402). This was continued for 44 days to establish the NAFLD model. The rats had an unlimited supply of food and water and were kept in a room with suitable temperature (22 ± 1°C), humidity (50–60%), and a 12-h light/dark cycle. The two diets were all purchased from Jiangsu Xietong Bio-engineering Co. Ltd. (Jiangsu, China).

### Sample Collection

After one night of fasting (44th day), all rats were sacrificed between the hours of 08:30 and 10:00. The parts of liver samples (middle lobe) were snap-frozen in liquid nitrogen and stored at −80°C for omics analysis and the making of frozen slices. Other parts of liver samples were fixed overnight in 4% formaldehyde for paraffin embedding. The blood samples were collected from the abdominal venous and placed in the tubes with sodium citrate (3.8%), centrifuged at 1,500 × *g* for 10 min to isolate serum and stored at −20°C.

### Serum Metabolites and Hormone

An automatic biochemical analyzer (type 7020; Hitachi; Tokyo; Japan) was used to measure the serum albumin (ALB), low-density lipoprotein (LDL), alkaline phosphatase (ALP), alanine transferase (ALT), aspartate transferase (AST), lactate dehydrogenase (LDH), high-density lipoprotein (HDL), and glucose (GLU) with commercially available kits (Maccura Biotechnology Co. Ltd., Sichuan, China). Serum total cholesterol (TCHO), triglyceride (TG), non-esterified FAs (NEFA) were measured using kits from Nanjing Jiancheng Bioengineering Institute (Cat.#A111-1-1, Cat.#A042-2-1, Cat.#A110-1-1, respectively). Moreover, insulin, growth hormone, and glucagon concentrations were determined by kits from AngleGene (i.e., Cat.#ANG-E21417M, Cat.#ANG-E21639M, and Cat.#ANG-E21597M).

### Histological Examination

Paraffinized liver sections were stained using hematoxylin and eosin (H&E). The H&E staining was used to calculate the steatosis, activity, and fibrosis (SAF) score to confirm the state of the fatty liver (15). The frozen liver sections were stained with Oil Red O for hepatic lipid accumulation analysis.

### RNA Extraction and Transcriptomic Analysis

**Figure 1:**
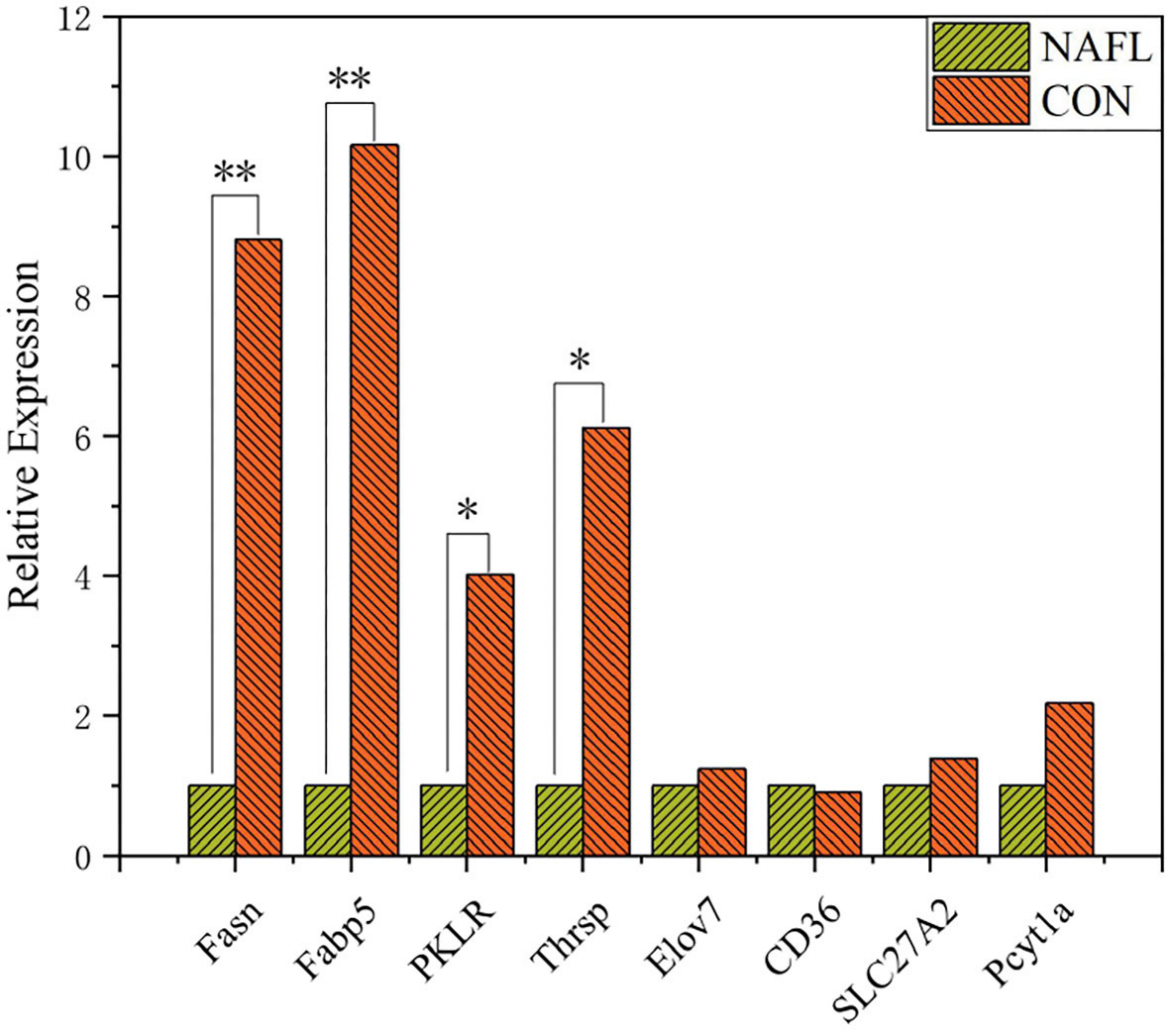
Validation of genes expression through RT-qPCR (*n* = 4). *Means *p* ≤ 0.05, **means *p* ≤ 0.01.

Liver RNA was extracted using Trizol (Life Technologies) and quantified using a fluorometric (Qubit 2.0, Thermo Fisher). The integrity of RNA was detected using Agilent 2100 bioanalyzer. Construction of the cDNA and sequencing were performed in Wuhan Metware Biotechnology Co., Ltd (Wuhan, China). The RT-qPCR of some genes was performed to confirm the reliability of RNA-seq data (Figure 1).

### Un-Targeted Lipidomic Analysis

Lipidomic analysis was performed using the LC-MS by Wuhan Metware Biotechnology Co., Ltd. (Wuhan, China). For lipid extraction, the liquid-liquid methyl tert-butyl ether (MTBE) lipid extraction method was used (16). The samples were briefly thawed on ice and the 20 mg samples were homogenized with a 1 ml mixture (including methanol, MTBE, and internal standard mixture) and a steel ball. The steel ball was taken out then the mixture was whirled for 15 min. Exactly 200 μl of water was added and the mixture was whirled for 1 min, then centrifuged at 4°C with 12,000 rpm for 10 min. Then, 300 μl supernatant was extracted and concentrated to powder. The powder was dissolved with 200 μl mobile phase B, then stored at −80°C for liquid chromatography-mass spectrometry (LC-MS) analysis.

The dissolved powder were analyzed using a liquid chromatography-electrospray ionization-mass spectrometry (LC-ESI-MS/MS) system (UPLC, ExionLC AD). The analytical conditions included, UPLC: column, Thermo Accucore™ C30 (2.6 μm, 2.1 mm^*^100 mm); solvent system, A: acetonitrile/water (60/40, V/V, 0.1% formic acid, 10 mmol/L ammonium formate), and B: acetonitrile/isopropanol (10/90 V V/V, 0.1% formic acid, 10 mmol/L ammonium formate). The effluent was alternatively connected to an ESI-triple quadrupole-linear ion trap (QTRAP)-MS. Linear ion trap (LIT) and triple quadrupole (QQQ) scans were acquired on a triple quadrupole-linear ion trap mass spectrometer (QTRAP) and QTRAP® LC-MS/MS System, equipped with an ESI Turbo Ion-Spray interface, operating in a positive and negative ion mode and controlled by Analyst 1.6.3 software (Sciex).

### Statistical Analysis

The insulin resistance level of homeostatic model assessment (HOMA-IR) (17, 18) was calculated for each rat using the following equation:


$$\begin{array}{l} \text{ HOMA}-\text{IR }=\\ \frac{\text{fasting blood glucose }\left(\text{mmol}/\text{L}\right)\;\times \text{ fasting insulin }\left(\text{mU}/\text{L}\right)}{22.5} \end{array}$$

The Principal Component Analysis (PCA), Pearson's Correlation Coefficient, Partial Least Squares-Discriminant Analysis (PLS-DA), and all other analyses were performed using R software (3.5.0).

DESeq2 was used to analyze the differentially expressed genes, Benjamini-Hochberg was used to control the False Discovery Rate (FDR), and PLS-DA was used to analyze the differentially expressed metabolites.

The principle for the selection of the differentially expressed genes is as follows,


$$\begin{array}{l} |\text{lo}{\text{g}}_{2}\text{Fold Change}|\;\geq 1,\text{ and False Discovery Rate }\left(\text{FDR}\right)\;<\;0.05. \end{array}$$

The principle for the selection of the differentially expressed metabolites is as follows,


$$\begin{array}{l} \text{fold change }\geq 2\text{ or }\leq 0.5\text{ and variable importance in projection}\\ \left(\text{VIP}\right)\;\geq 1. \end{array}$$

The number of rats for lipidomic: NAFL group was eight while CON group was six. For transcriptomic: both groups were of five which were randomly selected from the rats in each group. Other detections were the same as the lipidomic.

## Results

### The SAF Score of the NAFLD Model

**Figure 2:**
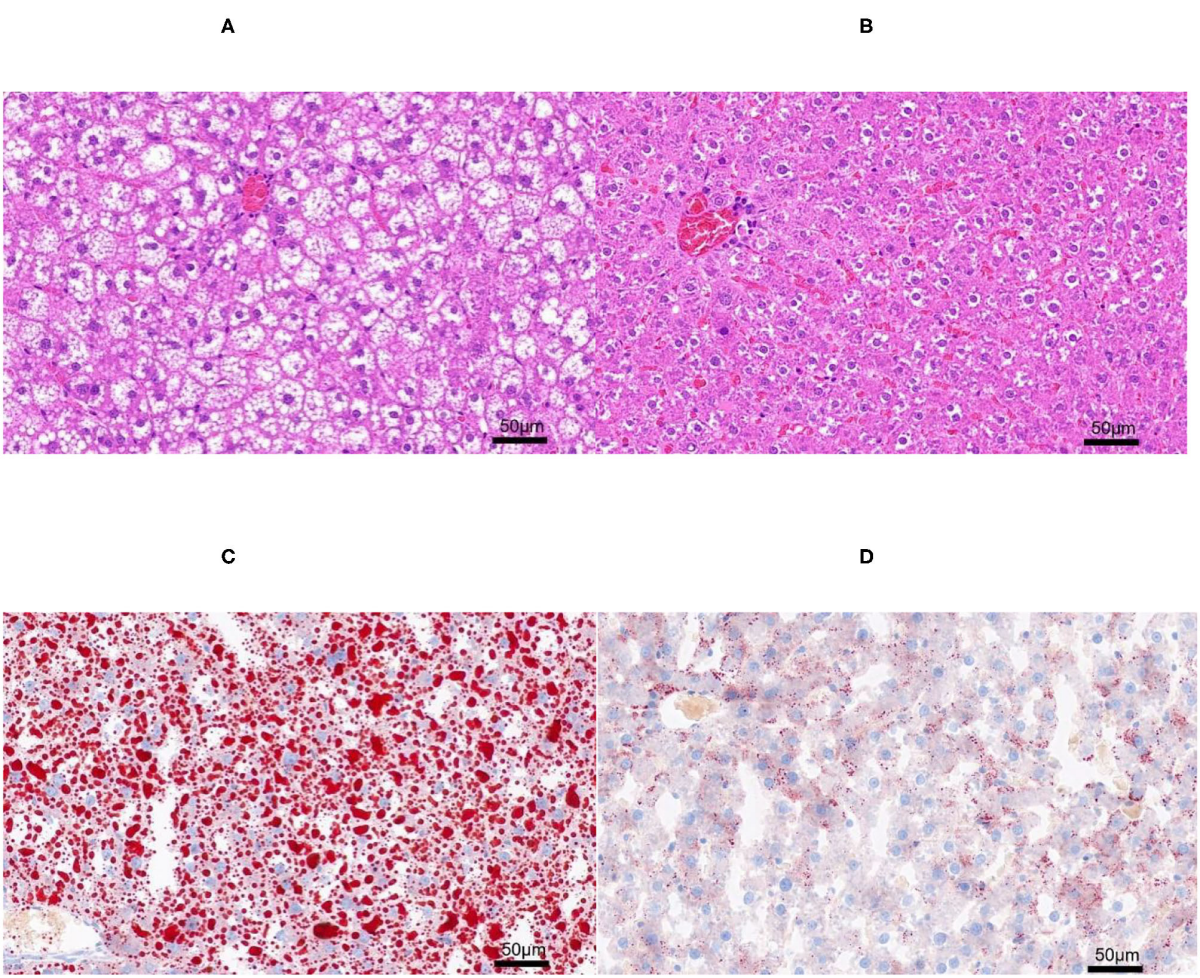
The H&E and Oil-red-O staining. **(A)** Non-alcoholic fatty liver (NAFL) group H&E staining; **(B)** control (CON) group H&E staining; **(C)** NAFL group Oil-red-O staining; **(D)** CON Oil-red-O staining.

The H&E and Oil-red-O staining were performed in both NAFL and CON groups (Figure 2) to determine the establishment of fatty liver model. Based on the scoring rules of SAF scores (15), the liver of the two groups did not reveal any Lobular inflammation. Thus, the rats in this experiment did not have a NASH. The rats in the NAFL group showed 5–33% of hepatocytes with large and medium-sized intracytoplasmic lipid droplets without the presence of enlarged hepatocyte of normal cells, no signs of fibrosis and inflammation. Therefore, the rats in the NAFL group had mild NAFLD, which was at the early stage of non-alcoholic fatty liver disease.

### Changes of Serum Metabolites and Hormones in Liver Steatosis Rats

The early stage of NAFLD rats showed higher serum concentration of NEFA (*p* = 0.0015), TG (*p* = 0.041), LDL (*p* = 0.0029), TCHO (*p* = 0.066), ALP (*p* = 0.0059), Insulin (*p* = 0.017), and glucagon (*p* = 0.026). However, those of the growth hormones (*p* = 0.012) and IGF-1 (*p* = 0.022) were lower. As for the GLU, HDL, ALB, AST, ALT, and LDH, no significant differences were noted between the NAFL group and the CON group. After calculating the HOMA-IR, the results of the NAFL group were significantly higher than that of the CON group, hence the insulin resistance level was higher (Table 1).

**Table 1:** Biochemical analysis of serum.

**Index**	**NAFL**	**CON**	**SEM**	***P*-value**
NEFA, mmol/L	0.41	0.29	0.02	0.0015
LDL, mmol/L	0.32	0.22	0.02	0.0029
ALP, U/L	464.75	277.17	39.61	0.0059
Growth hormone, ng/L	64.33	71.52	2.01	0.0120
Insulin, mU/L	5.37	3.84	0.39	0.0170
IGF-1, mg/L	2.62	3.17	0.15	0.0220
Glucagon, ng/L	41.76	35.84	1.65	0.0260
TG, mmol/L	1.59	1.17	0.13	0.0410
TCHO, mmol/L	2.82	2.23	0.21	0.0660
GLU, mmol/L	8.86	8.46	0.34	0.4100
HDL, mmol/L	1.49	1.33	0.14	0.4297
ALB, g/L	21.81	22.25	0.41	0.4600
AST, U/L	137.88	158.00	21.29	0.5200
ALT, U/L	43.50	41.83	2.86	0.6900
LDH, U/L	1197.63	1179.83	233.80	0.9600
HOMA-IR	2.23	1.44	0.17	0.0075

*p-value is from T-test by R (3.5.0). p-value ≤ 0.05 means statistically significant*.

### Fewer Genetic Changes Caused Huge Changes in Lipids

**Figure 3:**
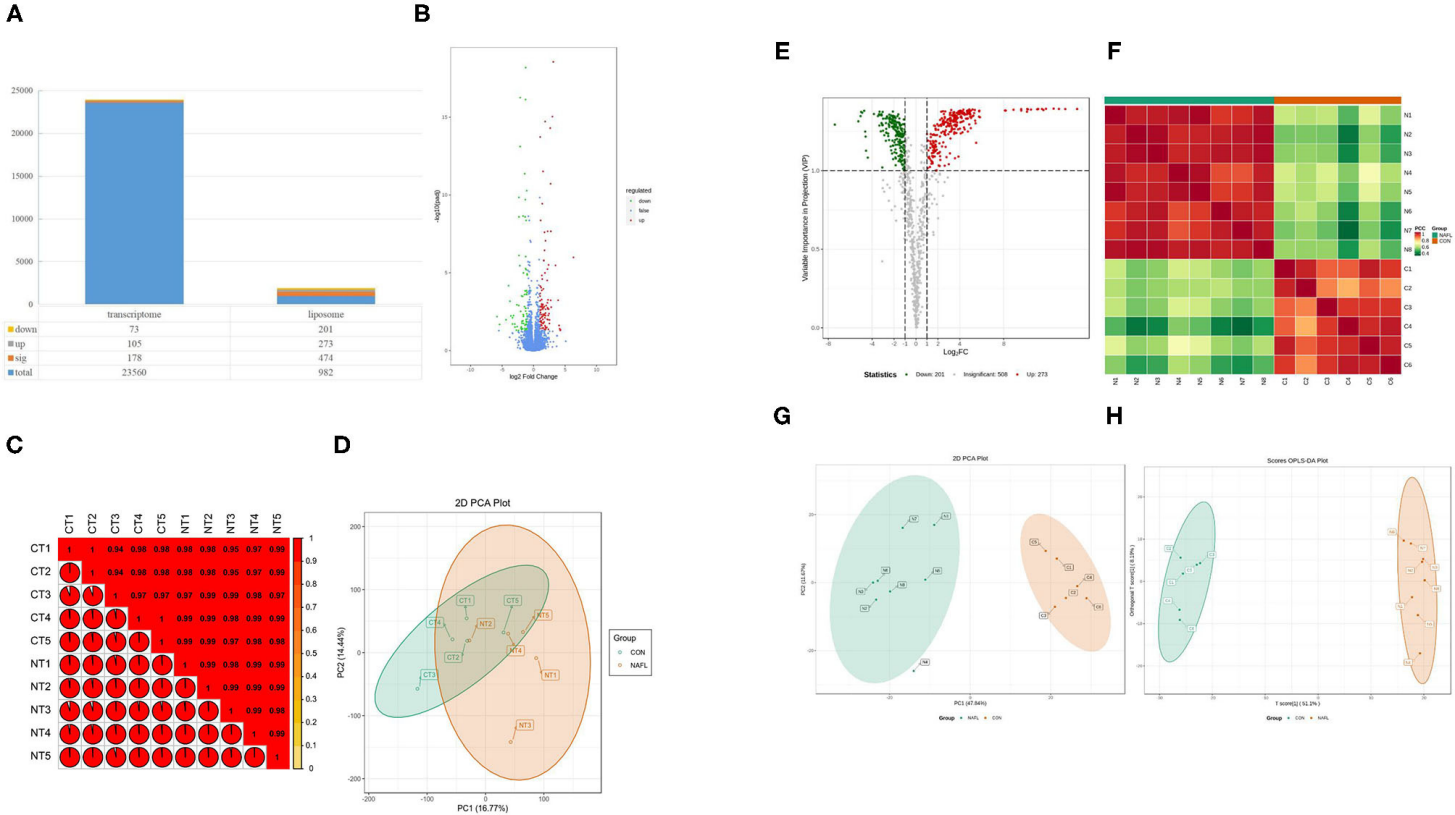
Fewer genetic changes caused huge changes in lipids. **(A)** The significance performance of the transcriptomic and lipidomic analysis. Down, up, sig, total mean the number of downregulated, up-regulated, significant, total detected genes or lipids; **(B)** Volcanic plot of differentially expressed genes; **(C)** Gene heat map of correlation between samples; **(D)** Principal component analysis (PCA) plot of genes; **(E)** Volcanic plot of differentially expressed lipids; **(F)** Lipid heat map of correlation between samples; **(G)** PCA plot of lipids, it should be noted that N4 is outside the circle; **(H)** OPLS-DA plot of lipids. CT1–CT5: the CON samples for transcriptomic, NT1–NT5: the NAFL samples for transcriptomic, C1–C5: the CON samples for lipidomic, N1–N5: the NAFL samples for lipidomic.

The study conducted the transcriptomic and lipidomic analyses of the two groups of rats to investigate the expression differences of mRNA and lipids between the two groups. Transcriptome results showed that only 178 differentially expressed genes were detected between the NAFL group and CON group. Out of which 105 were up-regulated and 73 downregulated (Figures 3A,B). A correlation test on each sample was further conducted to evaluate the correlation between biological repeats and the differences between treatments (Figure 3C). Consequently, a high correlation was found between biological repeats within the group. However, the treatment did not show a significant difference between the two groups. The Principal Component Analysis (PCA) results showed similar results (Figure 3D); the NAFL and CON group could not effectively separate. Therefore, it was thought that the treatment caused a mild reaction to the transcriptome.

A total of 982 metabolites were detected in the lipidomic analysis. Significant differences were noted in 474 metabolites between the NAFL group and CON group, out of which 273 were up-regulated and 201 down-regulated (Figures 3A,E). Nearly half of the lipids showed significant differences. The correlation test showed a high correlation within the biological repeats. Nevertheless, there was a significant distinction between the two groups (Figure 3F). The two groups could also be well-separated in the PCA diagram (Figure 3G), suggesting significant differences between the two groups; PLS-DA diagram (Figure 3H) showed similar results.

### The Most Prominent Characteristics of Transcriptomic and Lipidomic Analysis in the Early Stage of Fatty Liver

To learn more about the occurrence of NAFLD, the top 10 components with the most significant expression differences were analyzed between the NAFL group and CON group in the transcriptome (Table 2). Among the top 10 genes with significant expression differences, 6 were up-regulated, namely, *Fam179a* [1, Differential ranking (DR)], *RGD1562392* (5, DR), *Cyp3a62* (6, DR), *Cyp7b1* (7, DR), *Slc16a10* (8, DR), and *Ptprn* (10, DR), while 4 were down-regulated, namely, *Prkcdbp* (2, DR), *Fabp5* (3, DR), *Enpep* (4, DR), *Lhx8* (9, DR).

**Table 2:** The top 10 genes with the lowest *p*-value.

**Gene Name**	**log2_**Fold Change**_**	***p*adj**
*Fam179a*	3.134	2.79 × 10^−19^
*Prkcdbp*	−1.257	6.51 × 10^−19^
*Fabp5*	−2.121	5.55 × 10^−17^
*Enpep*	−1.261	7.35 × 10^−17^
*RGD1562392*	2.995	9.10 × 10^−16^
*Cyp3a62*	1.836	1.90 × 10^−15^
*Cyp7b1*	2.638	5.04 × 10^−15^
*Slc16a10*	1.045	1.88 × 10^−14^
*Lhx8*	−2.13	7.69 × 10^−14^
*Ptprn*	1.503	3.04 × 10^−12^

*Padj is the false discovery rate, used the method of Benjamini–Hochberg. Padj ≤ 0.05 means statistically significant*.

**Figure 4:**
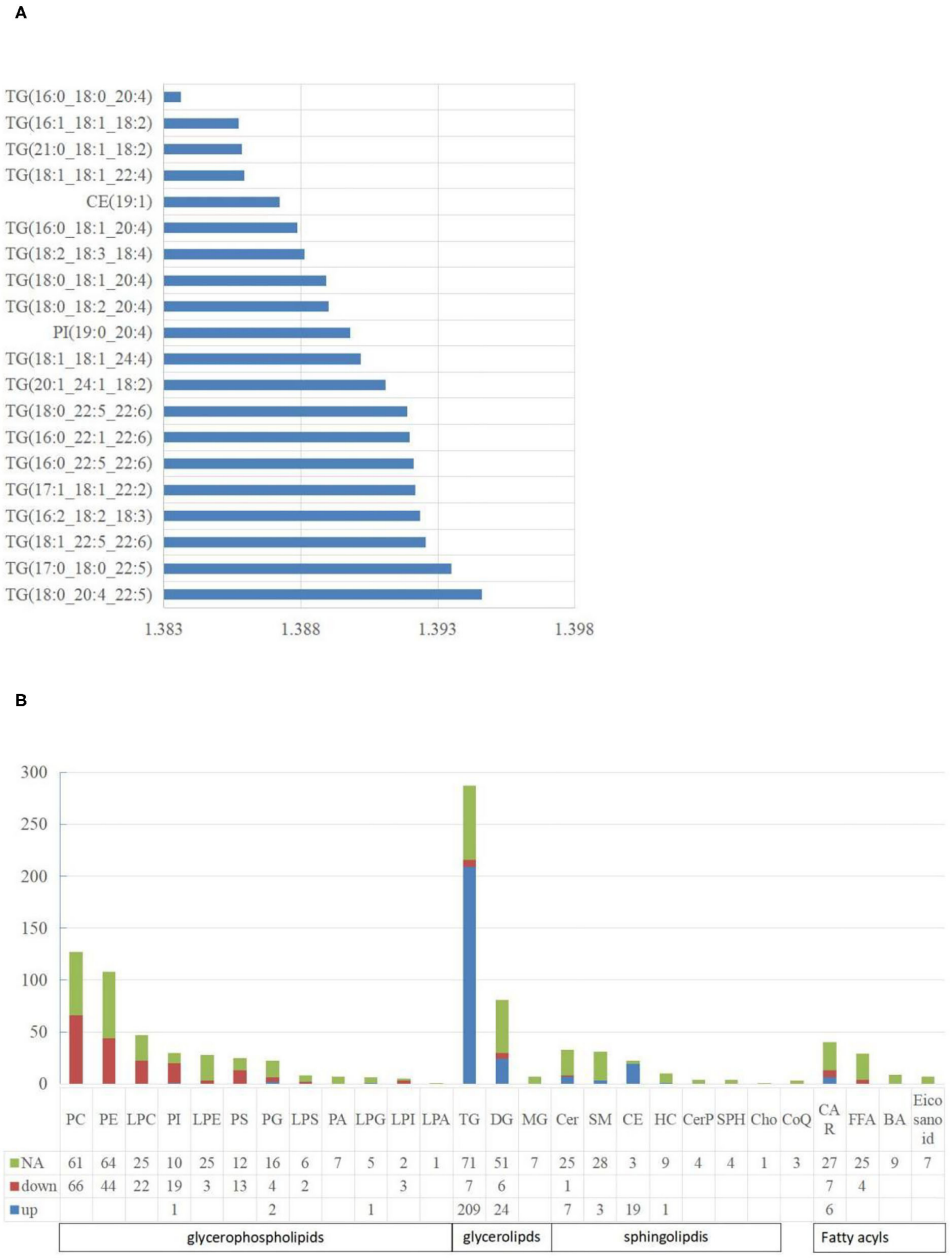
The characteristics of liposome. **(A)** The top 20 lipids with the highest VIP value. The abscissa is the VIP value; **(B)** The distribution of lipids in various classifications. NA means no significant differences, down means significantly down-regulated, up means significantly up-regulated. The abbreviations used in the study shows in Table 3.

For lipidomic analysis, the differentially expressed metabolites were screened out and the lipids were sorted based on the VIP value; the higher the VIP value, the more significant the difference. The top 10 lipids with the highest VIP value were all TGs (18 of the top 20 lipids were all TGs), and only the 11th and 16th were phosphatidylinositol (PI) and cholesterol esters (CE), respectively (Figure 4A). A total of 19 types of lipids (secondary classification) were detected and sorted based on the proportion of differentially expressed metabolites. The two most common types were TG (45%) and phosphatidylcholine (PC, 14%). Among all the metabolites detected, 287 types of TGs were detected, whereas 216 TGs were significantly different from those of the control group, out of which 209 were up-regulated and 7 were downregulated (Figure 4B).

### Characteristics of Each Classification of Liposome

A total of five types of lipids (primary classification) were detected. They included glycerol phospholipids (GP), glycerol lipids, sphingolipids, fatty acyl lipids, and isopentenol lipids. They were divided into several secondary classification lipids under each primary classification. For all the lipids detected, 48.27% of them in the NAFL group were significantly different from those in the CON group. Interestingly, 42.51% GP in the NAFL group significantly changed compared to that of the CON group. Ninety-seven-point seventy-eight percent (97.78%) of the lipids in these changes were downregulated and only four lipids were up-regulated including LPG (18:0/0:0), PI (19:0–20:4), PG (18:0–16:0), and PG (18:1–20:4). In contrast with the downregulation of most GP, 65.60% (246 species) of glycerides and 29.52% (31 species) of sphingolipids were significantly different, out of which 94.72% and 96.77% were up-regulated, respectively. Among the downregulated glycerides, there were seven types of TGs and six types of diglycerides (DGs). However, Cer (d18:1/14:0) was the only downregulated sphingolipid. Acyl carnitine (CAR), Non-esterified fatty acid (FFA), Bile acid (BA), and eicosanoids were classified as fatty acyl. Further, nine types of BA and seven types of eicosanoids were detected, with no significant difference. A total of 29 types of FFA were detected, out of which only four were significantly different, notably, and all of them were downregulated. A total of 40 types of CAR were detected, out of which 13 were significantly different. Six were significantly up-regulated and seven were downregulated. Only three kinds of coenzyme Q were detected in isopentenol lipids, and no significant difference was noticed between the two groups (Figure 4B).

**Table 3:** Abbreviations used in the study.

**Full name**	**Abbreviation**
Phosphatidylcholine	PC
Phosphatidyl ethanolamine	PE
Lysophosphatidylcholine	LPC
Phosphatidylinositol	PI
Lysophatidylethanolamine	LPE
Phosphatidylserine	PS
Phosphatidyl glycerol	PG
Lysophatidylserine	LPS
Phosphatidic acid	PA
Lysophatidylglycerol	LPG
Lysophatidylinositol	LPI
Lysophosphatidic acid	LPA
Triglyceride	TG
Diglyceride	DG
Monoglyceride	MG
Ceramide	Cer
Sphingomyelin	SM
Choiesl	CE
Glycosphingolipid	HC
1-Phosphate ceramide	CerP
Sphingosine	SPH
Cholesterol	Cho
Coenzyme Q	CoQ
Acyl carnitine	CAR
Non-esterified fatty acid	FFA
Bile acid	BA
Eicosanoid	Eicosanoid

### KEGG Enrichment Analysis of Differentially Expressed Genes or Lipids

**Figure 5:**
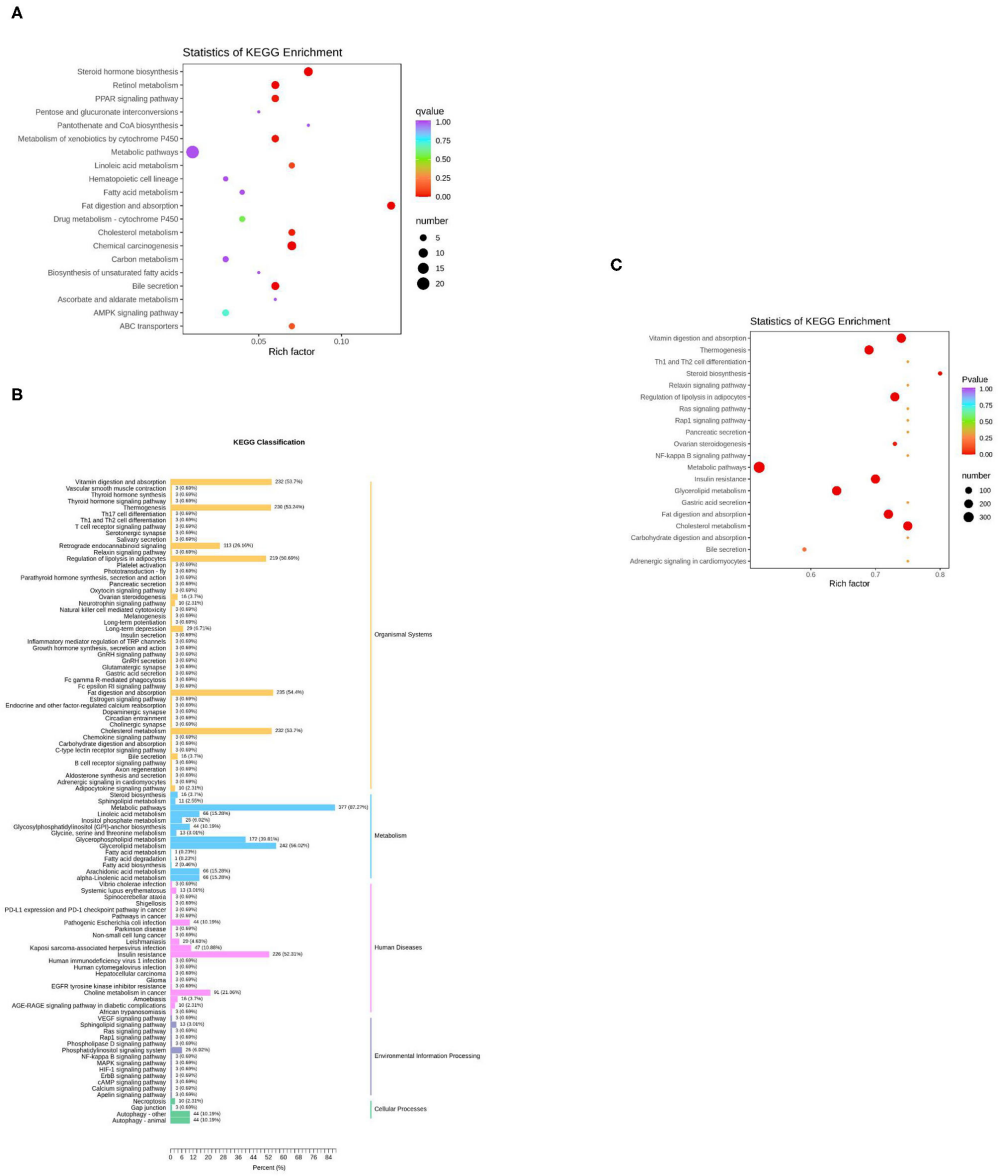
KEGG enrichment analysis of differentially expressed genes or lipids. **(A)** The top 20 pathways in rich factors of the gene pathway enrichment. Rich factor means the ratio of the number of differentially enriched genes to the number of annotated genes in this pathway, the number means the number of genes enriched in this pathway; **(B)** The KEGG enrichment of differentially expressed lipids; **(C)** The top 20 pathways in rich factor of the lipid pathway enrichment.

The KEGG function of differentially expressed genes was annotated, then a hypergeometric test was used to analyze the enrichment of the KEGG pathway of the differentially expressed genes. The top 20 pathways in rich factor are listed in Figure 5A, out of which only eight have a *q* ≥ 0.05, namely, steroid hormone biosynthesis, fat digestion and absorption, chemical carcinogenesis, retinol metabolism, bile secretion, metabolism of xenobiotics by cytochrome P450, peroxisome proliferator-activated receptor (PPAR) signaling pathway, and cholesterol metabolism.

For the lipids, KEGG results were presented in five parts (Figure 5B). In the part of Organismal Systems, five pathways (vitamin digestion and absorption, thermogenesis, regulation of lipolysis in adipocytes, fat digestion and absorption, and cholesterol metabolism) enriched 53.7, 53.24, 50.69, 54.4, and 53.7% of the differentially expressed metabolites, respectively. In the part of metabolism, two pathways (metabolic pathways, glycerolipid metabolism) enriched 87.27 and 56.02% of the differential expressed metabolites, respectively. In the part of human diseases, the insulin resistance pathway enriched 52.31% of the differential expressed metabolites. The Kyoto Encyclopedia of Genes and Genomes (KEGG) enrichment analysis of differential expressed metabolites was subsequently performed. The top 20 pathways in rich factor are listed in Figure 5C, out of which only seven have a *q* ≥ 0.05, namely, cholesterol metabolism, vitamin digestion and absorption, fat digestion and absorption, regulation of lipolysis in adipocytes, insulin resistance, thermogenesis, and glycerolipid metabolism.

### Joint Analysis of the Transcriptome and Liposome

**Figure 6:**
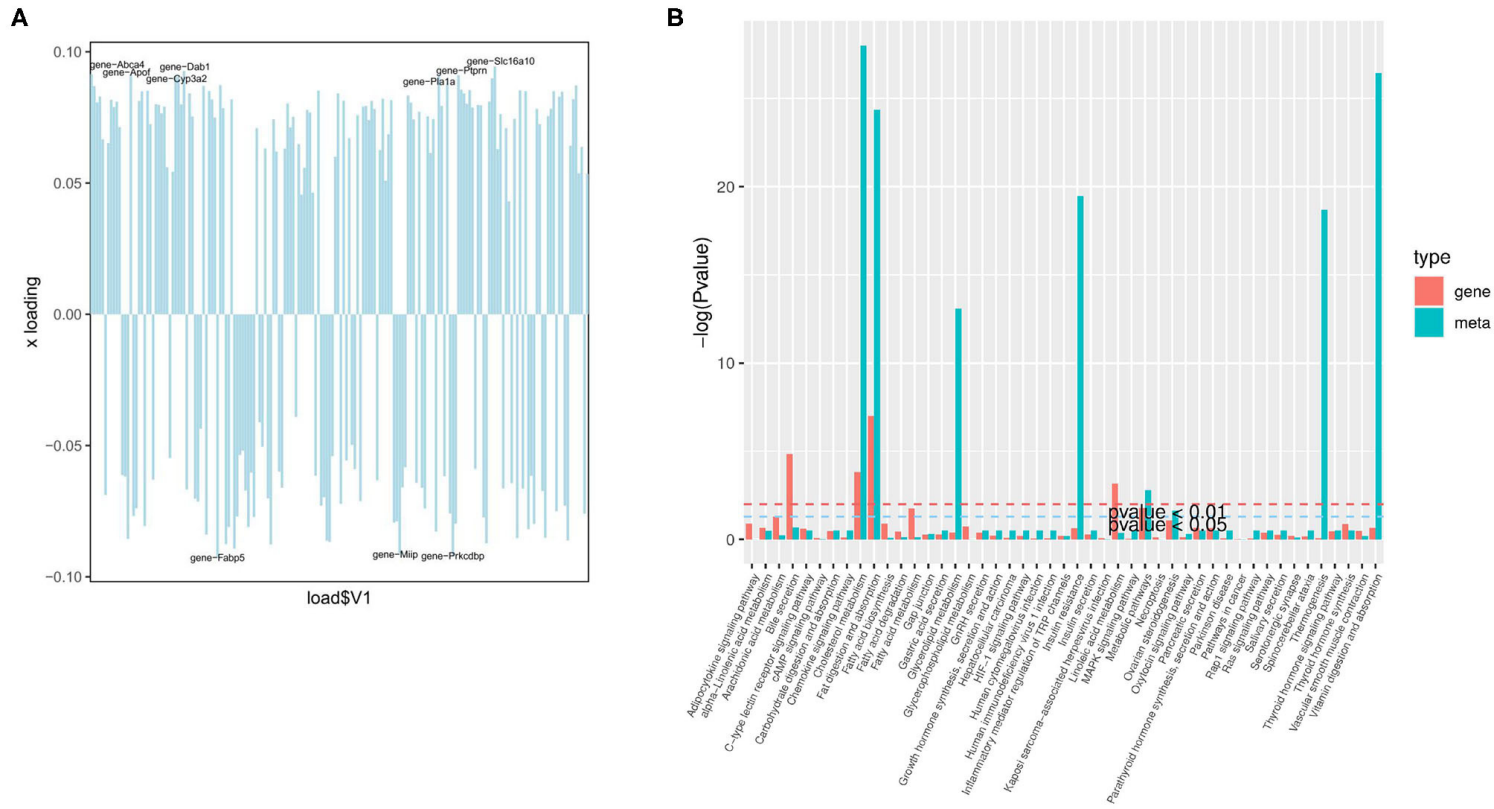
Joint analysis of the transcriptome and liposome. **(A)** Loading plot of genes; **(B)** The enrichment degree of pathways with both differentially expressed metabolites and differentially expressed genes.

The organism regulates metabolism by targeting the expression of genes (19). A joint analysis of transcriptome and liposome was conducted to identify the reasons for the differences between the two groups. First, O2PLS (two-way orthogonal partial least squares) was used to analyze the differentially expressed genes and metabolites. This was to identify the variables in one of the genes or metabolites with the greatest impact on the results of the other expressed by loading, in which the greater its absolute value, the greater the influence. The top 10 genes with the greatest impact on the results of the lipid group (Figure 6A) were screened. They included *Slc16a10* (up), *Dab1* (up), *Fabp5* (down), *Prkcdbp* (down), *Abca4* (up), *Apof* (up), *Ptprn* (up), *Miip* (down), *Pla1a* (up), and *Cyp3a2* (up). The four genes: *Slc16a10, Fabp5, Prkcdbp*, and *Ptprn*, were among the top 10 genes with the most significant differences.

Based on KEGG enrichment analysis of differentially expressed genes and metabolites, the pathways of common enrichment were screened including cholesterol metabolism, fat digestion and absorption, as well as metabolic pathways (Figure 6B). Therefore, changes in the absorption of lipids, the metabolism of cholesterol, and the metabolic pathway are the most prominent features in the early stage of NAFLD.

### The Early Stage of Fatty Liver Has Shown a Trend Toward NASH

Despite histological examination not showing any NASH representation in the two groups of rats (Figure 2), the expression of some genes and lipids related to NASH and fibrosis was explored to better understand NAFLD occurrence and development. The data of transcriptome and liposome of NAFL group showed some clues to the development of NASH. There were significant differences in the expression of some genes related to inflammation and fibrosis, namely, *Cxcl13* (up), *Nfatc4* (up), *Slit2* (up), and *Apoa4* (down). Furthermore, three kinds of lipids [DG (18:1–20:1), DG (18:1–20:0), and DG (18:0–20:4)] with significant up-regulated differences in expression were enriched in the NF-κB signaling pathway. Therefore, it was believed that in the rat fatty liver model, the early stage of fatty liver has shown a trend toward NASH.

## Discussion

Non-alcoholic fatty liver disease is a common chronic liver disease in China and western countries (17). Due to shortcomings in sample collection when investigating NAFLD in humans, this work used a rat fatty liver model. High-fat and choline-deficient diets were used to induce fatty liver model in rats, similar methods can reportedly induce NAFLD (14). By combining the H&E staining and Oil Red O staining results in liver sections, the rat early NAFLD model was successfully constructed. Many studies reveal that NAFLD increases the FFA, TG, LDL, TCHO, and ALP concentrations in the blood (20–24). The change of these blood indices is one of the typical characteristics of NAFLD. NAFLD is an excessive accumulation of lipids in the liver. The most prominent feature of these lipids is TGs (25, 26). The data from the study demonstrated similar findings in which about 72.82% of TGs were significantly up-regulated. The up-regulation of TGs during the occurrence of NAFLD is attributed to the synthesis rate of TGs (due to increased FAs uptake in the liver and esterification to TGs, and the DNL) in the liver exceeding the catabolic rate of TGs (which depended on FAs oxidation and the TGs output in the form of very-low-density lipoprotein) (10). Indeed, the synthesis and secretion of TGs did increase during this process. However, the output is insufficient to prevent steatosis (27). HOMA-IR data indicated that the NAFL group harbors a stronger insulin resistance than the CON group, and KEGG enrichment analysis results of liposome data showed that the Insulin resistance (*p* ≤ 0.001) pathway was also significantly enriched. Studies have demonstrated that insulin resistance increases the DNL process in the liver (28). Nevertheless, the results of the study did not show any evidence that the DNL process increased. The key genes *FASN* (FAs synthase) and *SCD* (Stearoyl-CoA Desaturase) in the DNL (29, 30) were significantly downregulated in the findings. Strikingly, studies have shown that lower levels of growth hormone during the occurrence of NAFLD promote the DNL process in the liver (14, 31). In the study, growth hormone significantly decreased in the NAFL group. Therefore, it is believed that the DNL process was downregulated in the study. In most studies like in the above discussion, the DNL process increases in NAFLD but may decrease in the NAFLD model induced by choline deficiency (32); moreover, the cause of this phenomenon warrants additional investigations.

Fatty acids intake depends on plasma FAs availability and FAs transport capacity (4). Several studies report that the content of free FAs in plasma increases in patients with NAFLD (24, 33). This is in line with the results. In this experiment, the availability of FAs of the NAFL group can be guaranteed because of the high-fat diet. The transport capacity of FAs also showed a significant increase. In the rat model of hepatic steatosis, *CD36* level was positively correlated with TGs content in the liver (34). In NAFLD patients, the CD36 protein increased (35). Liver *FABPs* promotes the shuttle of FAs, and its expression is positively correlated with NAFLD progress (36). Caveolins are a vital part of caveolae (transfers the FA) (37). *CD36, FABPs*, and *Caveolins* mediate the uptake of FA in Circulation on the Cell membrane (3). *Slc25a2* also regulates the uptake of circulating lipids (38). These findings demonstrate that the genes *CD36, FABP2, Slc25a2*, and *CAV2* in the NAFL group were significantly up-regulated compared to the CON group. Thus, it is concluded that the liver uptake of FA from blood was significantly increased in the NAFL group, hence, the content of TGs in the liver increased. However, the *FABP5* of the NAFL group significantly decreased, a phenomenon that seemingly occurs only in males (32), but the reason behind this remains unknown. Noteworthy, not all TGs and DGs in the NAFL group increased compared to the CON group. Seven TGs and six DGs decreased, and the reasons behind the decrease warrants further investigations.

Phosphatidylcholine is the most abundant phospholipid in all mammalian cell types and subcellular organelles. In rodents, about 30% of phosphatidylcholine (PC) biosynthesized in the liver is derived from the phosphatidylethanolamine N-methyltransferase (PEMT) pathway. The remaining 70% of PC is generated by the CDP-choline pathway (39). That is, 70% of PC originates from dietary choline, and a lower choline diet lowers the PC levels in the liver (40). Notably, choline lacked in the treatment, thus it is not difficult to explain the decrease of PC content. This might also be the reason for the accumulation of lipids in the liver caused by choline deficiency. A choline-deficient diet causes liver steatosis in rodents and humans, at least part of the reason is the decreased secretion of very-low-density lipoprotein (39). Nonetheless, evidence shows that a decrease in liver PC has been observed in human liver steatosis without choline deficiency (33). Low PC level is possibly attributed to other reasons other than the lack of choline. Notably, phosphatidylethanolamine (PE) is the second most abundant phospholipid in a mammalian membrane (39) and is also the source of the other 30% of PC. Liver endoplasmic reticulum (ER) stress increases in patients with NAFLD (10). In this experiment, the NAFL group may also have had ER stress. In NAFLD, endoplasmic reticulum (ER) stress is induced along with the cellular fat accumulation, then the level of PE decreased (41). Generally, liver liposome studies indicate a decrease in GP in patients with NAFL, including PC, PE, PS, and PI. In some cases, this change is only evident in the NASH, notably, these GPs were reduced in the present case. This work proposed an explanation for the decrease of PS. *Pla1a* (Phospholipase A1 Member A) in NAFL group was up-regulated, and the protein encoded by this gene is a phospholipase that hydrolyzes PS (42).

After NAFL occurrence, a few sphingolipids were also significantly disrupted. Changes in intracellular sphingolipid metabolism induced by excessive FFA potentially regulate NAFLD (43). In NAFL, ceramide promotes the uptake and esterification of free fatty acids in the liver and promotes lipid accumulation (44). Nevertheless, the FFAs with significant differences in the data were significantly downregulated, and this is possibly due to the fast esterification rate of FFAs. Reports indicate that ceramide induces insulin resistance (45). In the data, the ceramides had differences between the two groups wherein seven were up-regulated and one was downregulated. No significant difference in cholesterol was noted between the two groups. However, 86.4% of cholesterol esters significantly changed and all were up-regulated; only CE (14:0), CE (16:1), and CE (20:5) did not change. The reason for the significant increase of cholesterol esters is potentially due to the significant upregulation of the *Apof* gene which is related to cholesterol esterification and cholesterol ester transport (46, 47). Only one of the glycosphingolipids [SHexCer (d18:1:/24:0)] was up-regulated—a scenario yet to be reported. Acyl carnitines significantly changed, but the result was not uniform (17, 48, 49).

Among the top 10 genes with significant differences, only two genes were reported previously which showed different expression in NAFLD patients, namely, *Fabp5* (50) and *Enpep*. However, *Enpep* expression in a previous study (51) was significantly up-regulated in severe fatty liver than in the mild fatty liver, which was significantly downregulated in the NAFL group in this study. *Cyp3a62* is related to glucose and lipid metabolism. Its expression was significantly up-regulated in the NAFL group, which was also significantly up-regulated in the rats with a high-fat diet (52). One study reveals that after estrogen receptor α knockout, *Cyp7b1* expression was up-regulated and lipid deposition was increased (53). *Lhx8* expression was significantly downregulated in adipogenic progenitor cells of obese individuals (54). *Prkcdbp* was significantly downregulated when liver cirrhosis manifested (55). *In vitro* impacts of *Prkcdbp* loss include induction of Warburg metabolism, accelerated cell proliferation, and resistance to apoptosis (56). *Ptprn* was significantly up-regulated and potentially regulate the secretion of insulin and other hormones (57). *Slc16a10* is a transport carrier of aromatic amino acids (58). Nonetheless, *Fam179a*, which ranks No. 1, has no published research showing any relationship with the fatty liver and limited literature has shown that it is carcinogenic. Only one literature shows the evidence of that *Fam179a* in peripheral blood has a connection with the prediabetes. When pre-diabetic subjects with dyslipidemia, the expression of *Fam179a* significantly downregulated compared with normal subjects (59). Therefore, further research would be necessary to investigate the role of *Fam179a* plays in NAFLD.

As for the top 10 genes with the greatest impact on the results of the lipidomic. ABC transporters mediate lipid transport (60), and *Abca4* may also be involved in lipid transport. *Apof* was reported to have a connection with cholesterol esterification and cholesterol ester transport (47). The protein encoded by *Pla1a* is a phospholipase that hydrolyzes PS and lysophosphatidylserine (42). *Cyp3a2* was upregulated in the rat model of fatty liver which could promote 4 β-hydroxycholesterol production (61). Dab1 (encoded by *Dab1*), a Reelin adaptor protein is reported have connection with lipoproteins uptake (62). The most interesting thing was that studies have shown downregulation apoER2 or inhibition of Dab1 activity attenuated reelin and apoE (one of the apolipoproteins like Apof)-induced ABCA1(one of the ABC transporters like Abca4) expression. Reelin or apoE could enhance cholesterol efflux and up-regulate Abca1 expression via activation of the Dab1-PI3K-PKCξ-Sp1 signaling cascade (63).

Moreover, it was considered that in the early stage of fatty liver, a trend toward NASH is shown. *NFATc4* is one of the key transcription factors that control pro-inflammatory cytokine expression for adaptive immunity in T and B lymphocytes (64). Its activation promotes liver inflammation and fibrosis (65). The *SLIT2* can aberrantly regulate the inflammation in different inflammatory diseases and cell types (66). *Slit2* over expressed mice were much more vulnerable to CCl4-induced liver injury and more easily developed liver fibrosis (67). CXCL13 is a differentiation and hypoxia-induced adipocytokine that exacerbates the inflammatory phenotype of adipocytes through PHLPP1 induction (68). According to the experimental results, the expressions of *Cxcl13, Nfatc4* and *Slit2* were significantly up-regulated in the NAFL group. ApoA4 (encoded by *ApoA4*) has a variety of physiological functions, including anti-inflammatory and antioxidant activity, and circulating ApoA4 level has been used as a biomarker for the early diagnosis of liver fibrosis (69). The expression of *ApoA4* was significantly downregulated. A previous review establishes that inflammation is connected to the initiation of liver injury and the progression of NAFL to NASH in which the NF-κB signaling pathway plays a crucial role in this process (8). Therefore, it is believed that, in the early stage of fatty liver, the liver had shown the characteristics of NASH.

## Conclusion

In conclusion, the early stage of the NAFLD model of rats induced by high fat and choline-deficient diet reveals that a small amount of genetic changes potentially triggers a strong response to lipid components. A significant increase in fatty acids uptake and metabolism accompanied by cholesterol metabolism is the most prominent metabolic features of the liver in the early stage of NAFLD. In the early stage of fatty liver, the liver had shown some of the characteristics of NASH.

## Data Availability Statement

The datasets presented in this study can be found in online repositories. The names of the repository/repositories and accession number(s) can be found in the article/supplementary material.

## Ethics Statement

The animal study was reviewed and approved by Animal Welfare and Health Committee of Shandong Agricultural University.

## Author Contributions

RZ, WL, ZH, and SL conceived the project and designed the protocol. RZ, LF, YZ, and WL performed the experiments. RZ and WL wrote the manuscript. All authors read and approved the final manuscript.

## Funding

This study was funded by the National Natural Science Foundation of China, grant number is 31772624.

## Conflict of Interest

The authors declare that the research was conducted in the absence of any commercial or financial relationships that could be construed as a potential conflict of interest.

## Publisher's Note

All claims expressed in this article are solely those of the authors and do not necessarily represent those of their affiliated organizations, or those of the publisher, the editors and the reviewers. Any product that may be evaluated in this article, or claim that may be made by its manufacturer, is not guaranteed or endorsed by the publisher.
